# An audiological perspective on ‘‘Two further patients with Warsaw breakage syndrome. Is a mild phenotype possible?”

**DOI:** 10.1002/mgg3.1283

**Published:** 2020-06-05

**Authors:** Davide Brotto, Renzo Manara, Pietro Scimemi, Flavia Sorrentino, Silvia Montino, Francesca Maritan, Ezio Caserta, Elisa Lovo, Alessandro Martini, Rosamaria Santarelli, Patrizia Trevisi

**Affiliations:** ^1^ Neurosciences Department University of Padova Padova Italy; ^2^ ENT Unit Azienda Ospedaliera Università di Padova Padova Italy; ^3^ Neuroradiology Neurosciences Department University of Padova Padova Italy; ^4^ Audiology Service Ospedale Santi Giovanni e Paolo Venezia Italy

To the Editor

We read with interest the article by Bottega et al. entitled ‘‘Two further patients with Warsaw breakage syndrome. Is a mild phenotype possible?” (Bottega et al., 2019) suggesting the existence of a less severe phenotype of the syndrome. The very same patients were evaluated in our center for the management of the hearing loss and something concerning these specific aspects can be added to the nice description of the abovementioned article.

First of all, high‐resolution temporal bone CT revealed in both sisters the presence of cochlear malformation characterized by bilateral cystic aspect of the middle and apical turns consistent with incomplete partition type 2 without enlarged vestibular aqueduct (Sennaroğlu & Bajin, 2017). In spite of a similar cochlear malformation, the older sister presented a moderate progressive hearing loss with current good benefit with hearing aids, while the younger one presented congenital profound hearing loss that required bilateral sequential cochlear implantation. Both CT morphology and the hearing performance should be added to Warsaw syndrome phenotype as previous characterizations do not match with this precise description (Alkhunaizi et al., 2018).

In addition, while the first sister presented with a normal speech and language development, the second presented a severe delay. Concerning this specific aspect, there is something to elaborate on: the child with speech and language development delay was extremely reliable in term of psychoacoustic feedbacks for the fitting of the cochlear implants. The ability to discriminate the difference in pitch and loudness seemed to be present when stimulation was provided from different electrodes of the array. ECAP values, tested via AutoNRT® (Cochlear®), provided a great underestimation of the C levels tested by mean of psychoacoustic technique (30–40 Cl difference between the ECAP and the C levels). The difference was so big that T levels in some electrodes, at the end of the fitting, were higher than the ECAP values, thus suggesting that previous fittings based on ECAP values most probably were not adequate to provide the best hearing outcome. The borderline cognitive results reported by Bottega et al. (2019) are not sufficient to explain the speech and language development delay, especially given the good auditory results at audiological testing by mean of play audiometry after sequential cochlear implantation (first cochlear implant performed at age 1.5 on the right side and second at 4 years old, aided PTA 32.5 dB nHL on the right and aided PTA 50 dB nHL on the left) that are appropriate for a normal speech and language development.

Moreover, qualitative evaluation of ECAP responses, in terms of latency and shape of the action potential generated by the electric stimulation, although not conclusive, seemed to suggest that also auditory neuropathy can be excluded, at least for the intracochlear branch of the auditory nerve. Consequently, the cochlear nerves cannot be considered pathological and, at least, the peripheral electrical stimulation is efficient, while the speech discrimination may be not. In addition, previous studies demonstrated that even in case of cochlear nerve deficiency the outcome of cochlear implantation can be good (Brotto et al., 2019). As all the reported data seem to show that the presence of the bilateral malformation does not impair the hearing outcome after cochlear implantation, they also suggest more likely a disorder affecting the central nervous system with a possible relationship with the reduced cognitive performance. This observation seems to be in contrast to the previously reported impression that speech and language delay in other patients was probably due solely to the presence of the hearing loss (Alkhunaizi et al., 2018).

Finally, in the latest months, L.I.S. (Italian sign language) was introduced in her rehabilitative protocol by speech and language therapist. The introduction of sign language and the increase in auditory perception skills, due to the second cochlear implant and the new fitting of the first, favored the improvement of auditory attention, language production and general communication skills.

In conclusion, it should be noted that, strictly from an audiological point of view, despite the similar CT morphological findings of the temporal bones of both sisters, the auditory performance is extremely different, and the younger sister presented a severe audiological phenotype. Consequently, we should add that cochlear implantation is mandatory in case of profound hearing loss even in Warsaw syndrome, since good hearing results are reported in different syndromic patients. However, the outcome can be influenced by central nervous system disorders, causing underachievement in terms of speech development; so visual reinforcement should be taken into consideration in order to improve communicative skills in such patients. Patients may need the attention of several specialists in order to strengthen their psychosocial and communicative development.

As an in‐depth understanding of Warsaw syndrome has not been achieved yet, we believe that any additional data is precious for defining a more exhaustive phenotypic spectrum and attaining a better clinical assistance, even more from an audiological point of view.

## AUTHOR CONTRIBUTIONS

Davide Brotto and Patrizia Trevisi made substantial contributions to conception and design of the study. Renzo Manara, Pietro Scimemi, Rosamaria Santarelli, and Alessandro Martini involved in drafting the manuscript or revising it critically for important intellectual content. Flavia Sorrentino, Silvia Montino, Maritan Francesca, Ezio Caserta, Elisa Lovo involved in acquisition, analysis, and interpretation of data.

## CONFLICT OF INTEREST

No conflicts to declare.

## DATA AVAILABILITY STATEMENT

The data that support the findings of this study are available on request from the corresponding author.
